# Phenotype Ontology Research Coordination Network meeting report: creating a community network for comparing and leveraging phenotype-genotype knowledge across species

**DOI:** 10.4056/sigs.2926219

**Published:** 2012-07-20

**Authors:** Paula Mabee, Andrew Deans, Eva Huala, Suzanna E Lewis

**Affiliations:** 1 Pennsylvania State University, University Park, PA, USA; 2 Department of Plant Biology, Carnegie Institution for Science, Stanford, CA USA; 3 Department of Biology, University of South Dakota, Vermillion, SD, USA; 4 Genome Division, Lawrence Berkeley National Lab, Berkeley, CA, USA

## Abstract

Representing phenotype in a way that can be linked to thousands of molecular genetic and environmental databases is an unresolved research challenge. A recent meeting of the Phenotype Research Coordination Network (RCN) aimed to coordinate and leverage current efforts. The three day summit meeting was hosted by NESCent (The National Evolutionary Synthesis Center) in Durham, North Carolina on the 23^rd^ – 25^th^ of February, 2012.

## Introduction

Knowing how an organism looks, behaves and functions, i.e., its ‘phenotype’, is central to interpreting the interaction of underlying genes and environmental effects. The goal of the Phenotype RCN is to establish a network of experts, drawn from plant, animal, and other research communities, who are independently developing ways to represent phenotypic data and facilitate coordination across these multiple efforts. The Phenotype RCN fosters interactions among researchers by providing introductions, opening up new channels of communication, holding meetings, providing educational opportunities, supporting collaborative exchanges, and coordinating activities all aimed at advancing the field by: (1) developing standards and best practices for accurate phenotype representations; (2) building key reference ontologies for plants, vertebrates, and arthropods; and (3) cross referencing these ontologies so that key data can be easily accessed and shared.

## Why phenotypes?

Charles Darwin was the first to recognize that most ‘characters’ used to classify organisms and place them into the same group are shared because they were inherited from their common ancestor. Simply put, all organisms, including humans, share a common biological history and it is on this point that much of comparative biology, systematics, and translational research hinge. Phenotypes are manifestations of the underlying molecular network under the influence of this environment. Through the application of community standards that enable comparisons across different resources we can achieve several important goals, including:

1. Increased searchability, by uncoupling authors’ editorial style from queries.
2. Rapid identification of species (or greatly narrowed search space) using only observable characters and environment.
3. Quantitative comparison across species, so researchers can repurpose data collected in one organism to gain insight into the biology of other organisms.
4. Extraction of information from a digitized library of biodiversity knowledge
5. Translation of discoveries from basic science into medical practice and *vice versa* through searches that provide prioritized lists of candidate genes to guide further validation strategies in discovery projects.

Shared standards for describing phenotypes will provide a stable foundation for developing automated search and comparison algorithms, with rigorous statistical and semantic underpinnings to assure the results are as precise as possible. These search results can identify relevant genotype-phenotype associations, and thereby maximize the utility of data collected across all research systems. Translating information across biological systems and species boundaries is a significant research bottleneck. The need becomes more urgent when considering the advent of thousands of new genome sequences, facilitated by next-generation sequencing technologies. The RCN meeting aimed to move the community toward the goal of phenotypic information integration.

## Attendees

There were 61 participants, including representatives from the USA, Canada, France, Germany, UK, Switzerland, and Venezuela. Not only was the meeting geographically diverse, but the fields of study also ranged widely. Diverse taxonomic groups, including plants, vertebrates, arthropods, poriferans, and fungi were all represented, as well as cognitive scientists, bioinformaticians, biomedical researchers, population biologists, and semantic knowledge engineers. The intense interactions, the varied perspectives, and the venue (at the National Evolutionary Synthesis Center in Durham, NC) all created an ideal environment for concentrated and fruitful discussions.

Almost half the people attending were new to the field of ontologies and few participants had met prior to this meeting. The first day, therefore, was spent completing hands-on exercises in small groups, which gave participants a common experience with ontology building and phenotype annotation (using a customized Phenote interface). The common underlying principle, regardless of area of interest or granularity, is that all phenotypic descriptions can be decomposed into two parts: An entity that is affected, be it an enzyme, an anatomical structure or a complex biological process; and a quality of that entity. These simple exercises employed images of dogs and humans as a way to illustrate the complicated issues that arise, e.g., ‘What is a snout relative to a nose?’ and ‘Can the same anatomical ontology be used for two different species, e.g., dogs and humans?’ On the second day of the workshop we focused on the kinds of discoveries that could be made with phenotypic data through the use ontological descriptions. On the final day we again broke out into working groups, first organized along taxonomic lines, and then reorganized as challenge-centered groups (e.g., how to express complex and quantitative phenotypes).

## Highlights of Presentations

Gary Merrill (Ontolytics, LLC) gave an excellent presentation explaining exactly why “ontology is such a pain”. He used a classification of brass instruments as an example illustrative of the key issues and discussed why these issues matter, and how easy it is to introduce errors. For anyone familiar with the development of ontologies, these examples rang true. A major point was that it is essential to be as clear as possible in defining the ‘thing’ you are talking about. Bruce Kirchoff (UNC Greensboro) pointed out that definitions that describe the full range and extent of possible instances are cognitively much more understandable to humans (i.e., curators carrying out annotations) than definitions based on a single canonical archetype or type specimen (e.g., we should define *chair* by showing multiple images, all of which are representative of the class *chair—*beanbags included—rather than via a single image of a stereotypical 4-legged chair). Thus, ontology builders must bear in mind how human cognition works when they are constructing definitions; definitions should be descriptive of the range of possibilities rather than describing some hypothetical consensus ideal of that class. In the same vein, it is vitally important to remember that while we use terms (aka domain jargon) to refer to things, which particular term or name is used is unimportant (or only mildly important). It is the *meaning* of that term that is crucial, including both its definition and its relationship to other things. Gary also reminded the group that ontology builders and users must constantly bear in mind that an ontology is as much about how categories are related to one another as it is about defining these categories. In fact, it is the relationships that are used as logical definitions by software for reasoning. Well-constructed ontologies, with all classes logically defined, can serve as an algebra for answering questions and knowledge discovery. Gary also reminded everyone of the necessity of building toward a specific, practical purpose or usage. Whether for search and retrieval, or data integration, hypothesis generation, or some other purpose; knowing the kinds of questions the ontology will be used to answer is essential to for making design decisions and avoiding mission creep. Taking a pragmatic approach was a philosophy echoed repeatedly throughout the course of the meeting.

Melissa Haendel (Oregon Health Sciences University) summarized one of the most crucial needs in the community: effective organism-specific and cross-organism anatomy ontologies. Given that anatomists, comparative morphologists, developmental biologists, immunologists, neuroscientists, and other biologists all desire the ability to query for gene expression and phenotypes across species, what must the ontologists provide to capture existing knowledge computationally, and what must tool-builders provide as query engines and user interfaces? She noted that all of the different perspectives for classifying data are useful—by compositional parts, by function, by shape, through development, or by evolutionary history—and an anatomy ontology must support all of them. Both she and David Osumi-Sutherland (University of Cambridge), like Bruce earlier, are approaching anatomical classification from a modular standpoint. Melissa also reminded everyone that anatomy classes are core elements of many other ontologies, and are implicitly within the Gene Ontology, the Mammalian Phenotype Ontology and numerous others, and this leads to data silos. By developing core anatomical ontologies that are shared, these data silos can be integrated and this knowledge mined *in toto*. The idea is to modularize based on domain or taxon, import and reuse (rather than cross-referencing or “aligning”), and work together to distribute the total work.

A growing number of consortia and databases are sharing the same approach for describing phenotypes, e.g., Phenoscape, The Virtual Human Physiology project (VPH), the International Mouse Phenotyping Consortium, the Neurobiology Information Framework, Flybase, Dictybase, Wormbase, ZFIN, Mouse Genome Database (MGD), and other international projects. George Gkoutos (University of Cambridge) emphasized the power of a common representation of phenotype in gene discovery relevant at the clinical level. Jim Balhoff (NESCent) described the semantics of phenotype representation and some of the current limitations in reasoning.

Christopher Mungall (Lawrence Berkeley National Lab), Monte Westerfield (University of Oregon), Paula Mabee (University of South Dakota), Andy Deans (^1^Pennsylvania State University), and Sue Rhee (Carnegie Institution for Science) all ably summarized the need for logic-centered approaches, as contrasted to language-centered approaches, to capture phenotypic data and promote discovery. Chris noted that a logic-centered approach is in fact made easier because biology is modular, for example the phalanxes of the hand and foot are repeated units, both distally and across appendages. During a session on impact, each speaker pointed out that without these logic-centered data structures in place to support computer-based reasoning, activities such as querying, data mining, and data-driven hypothesis generation are impossible. Chris provided an excellent review of the currently available reasoners that operate on OWL2-DL. Paula pointed out examples of new research questions generated by data that the Phenoscape project has collected so far, such as: “Are gill rakers absent in eels because of changes in regulation of the *eda* pathway?” “Did the taxon *Mola* lose its caudal fin because of changes in regulation of *yap1*?” Andy pointed out the revolutionary impact the approach is having on his research in descriptive taxonomy; putting into a powerful queryable form the information from millions of analog descriptions, which contain tens of millions of natural language phenotype annotations and are distributed across thousands of journals. He noted that this is information that would otherwise be wasted. In the field of biodiversity there are now multiple new web sites available and new tools for automatic identification of species. Sue Rhee focused on what bio-ontologies can do for us, providing powerful approaches from her research on *Arabidopsis*, including methods to find all genes in a biological process and ways to model functions, processes, and phenotypes to explain traits and predict phenotypes from genotypes. The group overall was optimistic about the power a standard approach for data synthesis and discovery based on semantic phenotypic descriptions, and the potential for such an approach in making data accessible to very broad groups of researchers for new and synthetic research. We have a model for representing knowledge about phenotypes at a fine scale and in a semantic way. However more multi-species anatomy ontologies, refinements to the phenotype ontologies, improved reasoners, and more accessible annotation tools to address expressivity issues are all still urgently needed.

## Conclusion

The major thrust of the meeting was on identifying points of intersection and the immediate steps that could be undertaken following the workshop to lay the groundwork for the future. As documented in the working group reports, critical directions for new research were identified, and several collaborating groups were formed to moved ahead with prototyping or otherwise investigating opportunities for this work. In retrospect, a remarkable aspect of this meeting was the excitement it generated, an essential first step for building and coordinating collaborative efforts. Given the positive outcomes of this workshop, we are planning to gather again in the fall to follow up and build on the momentum this meeting generated. The Phenotype RCN is an open community that welcomes contributions from all researchers interested in computable representation of phenotypes.

